# Two remarkable serine/leucine polymorphisms in *Helicobacter pylori*: functional importance for serine protease HtrA and adhesin BabA

**DOI:** 10.1186/s12964-024-01635-5

**Published:** 2024-05-02

**Authors:** Steffen Backert, Nicole Tegtmeyer, Anselm H. C. Horn, Heinrich Sticht, Bodo Linz

**Affiliations:** 1 Department Biology, Division of Microbiology, Friedrich-Alexander Universität Erlangen-Nürnberg, Staudtstr. 5, Erlangen, 91058 Germany; 2 Division of Bioinformatics, Institute of Biochemistry, Friedrich-Alexander-Universität Erlangen-Nürnberg, Fahrstr. 17, Erlangen, 91054 Germany

**Keywords:** BabA, HtrA, Evolution, Adaptation, SNP

## Abstract

Single nucleotide polymorphisms (SNPs) account for significant genomic variability in microbes, including the highly diverse gastric pathogen *Helicobacter pylori*. However, data on the effects of specific SNPs in pathogen-host interactions are scarce. Recent functional studies unravelled how a serine/leucine polymorphism in serine protease HtrA affects the formation of proteolytically active trimers and modulates cleavage of host cell-to-cell junction proteins during infection. A similar serine/leucine mutation in the carbohydrate binding domain of the adhesin BabA controls binding of ABO blood group antigens, enabling binding of either only the short Lewis b/H antigens of blood group O or also the larger antigens of blood groups A and B. Here we summarize the functional importance of these two remarkable bacterial SNPs and their effect on the outcome of pathogen-host interactions.

## Background

*Helicobacter pylori* is a successful human pathogen known to colonize the stomachs of almost half of the world’s human population. The association of *H. pylori* with its human host is ancient and dates back over 100,000 years, and probably much longer [1]. *H. pylori* is naturally competent for the uptake and incorporation of exogenous DNA into its genome [2]. In addition to a high recombination rate, *H. pylori* has an exceptionally high mutation rate of approximately 2 x 10^-5^ changes per site per year that exceeds that of other bacteria by one to two orders of magnitude [3, 4]. As a result, strains of *H. pylori* exhibit a great genetic diversity that extends to both sequence diversity and gene content. While many virulence genes belong to the core genome, other virulence factors are variably present among strains [5]. Substantial allelic variation among the major *H. pylori* virulence factors drastically affects the pathogen-host interactions. The *cag* pathogenicity island (*cag*PAI) encodes a type IV secretion system (T4SS) that injects the oncoprotein CagA, ADP-heptose, and chromosomal DNA into eukaryotic cells. Inside the host cells, CagA is phosphorylated by host cell kinases at the tyrosine residues of the so-called EPIYA motifs, which then triggers a diverse cascade of intracellular signaling events that interfere with cellular functions and disrupt cell morphology [6]. These EPIYA motifs are usually present as three distinct repeats, EPIYA-A, EPIYA-B, and EPIYA-C or EPIYA-D, that differ from each other by their specific flanking amino acid sequences. While CagA from East Asian *H. pylori* contain EPIYA-A, -B, and -D, strains from other regions, the so-called Western strains, contain EPIYA-A, -B, and -C motifs. EPIYA-C can be present as one or multiple consecutive copies (ABC, ABCC, ABCCC, etc), but EPIYA-D is typically present only once [5]. East Asian CagAs were shown to induce stronger inflammation levels compared to CagA from Western strains [6]. Likewise, the virulence level of the vacuolating cytotoxin VacA, which induces the formation of cellular vacuoles from lysosomes, is determined by allelic variation, most notably in the N-terminal signal peptide region (alleles s1, s2), and in the middle region (m1, m2). While s2/m2 *vacA* strains are not cytotoxic, strains with s1/m1, and to a lesser degree s1/m2, *vacA* alleles cause marked development of cellular vacuoles and apoptosis, and interfere with cellular signaling pathways [7]. Allelic variation is also known from genes encoding many other virulence factors, including the structural *cag*T4SS component CagY and outer-membrane proteins such as HopQ and BabA. There are two distinct types of HopQ adhesins, of which type I is associated with virulent strains [8]. Variation in the carbohydrate binding domain (CBD) of the blood group adhesin BabA mediates differential binding to specific blood group antigens. Most *H. pylori* strains can bind each of the fucosylated blood group A, B, and O antigens and are regarded as generalist strains. In contrast, strains expressing BabA variants that specifically bind blood group O antigens with high affinity, but not A or B antigens, are known as specialist strains [9].

Besides gene variants that differ from other alleles by numerous changes, single nucleotide polymorphisms (SNPs) in many different genes were identified by several studies, including genome comparisons of isolates from chronic infection, natural transmission, and experimental infection experiments [3, 4, 10–13], and from genome-wide association studies (GWAS) [14–16]. However, the effects of individual SNPs on the bacteria, and specifically the pathogen-host interactions, were unknown. Two recent studies identified SNPs encoding a serine/leucine change in two *H. pylori* virulence factors, the serine protease HtrA [17] and the BabA adhesin [18], and unraveled in great detail how each of the amino acid changes influences the interaction of the bacteria with the host. Here, we discuss these remarkable analogous bacterial SNPs and how they impact the interaction of *H. pylori* with its human host.

### Functional relevance of a serine/leucine polymorphism in serine protease HtrA

**Fig. 1:**
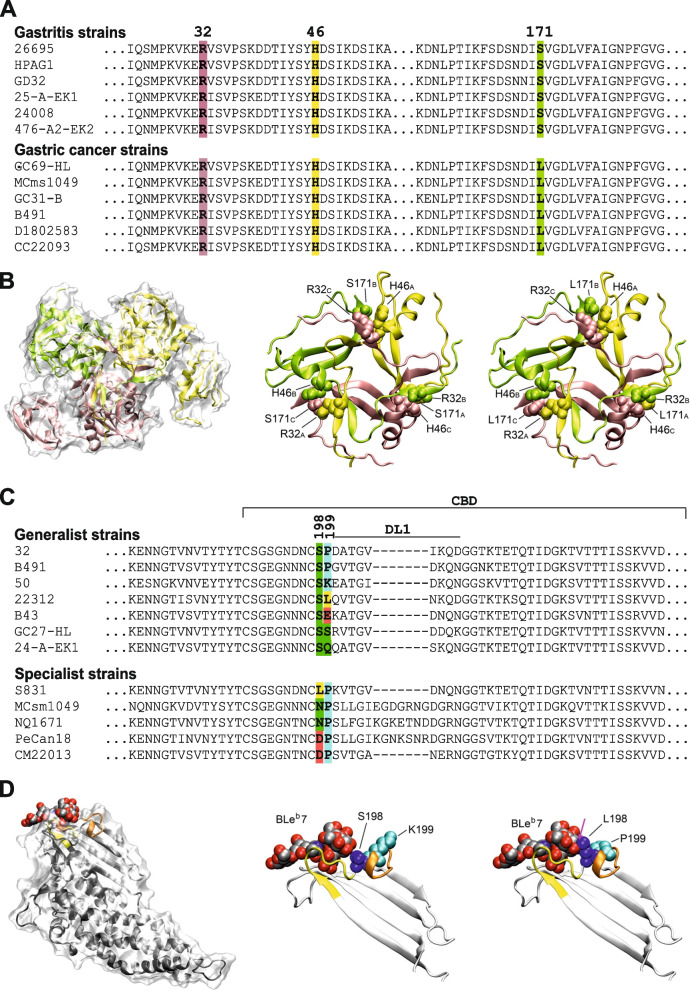
SNPs in *H. pylori* HtrA and BabA and their effect on protein structure. **A** The disease-related SNP at HtrA position 171 in strains from patients with gastritis or gastric cancer and amino acids R32 and H46 involved in trimer formation. **B** Effect of the S171L exchange on the HtrA trimer interface. (left) Structure of trimeric HtrA (PDB:5Y28) as transparent surface representation. The three subunits shown as yellow, green, and pink ribbon. (middle) Enlargement of the trimer interface for the S171 variant. Residues S171, R32, and H46 are shown in space-filled presentation and are labelled. Subscript letters denote the origin from the three different subunits. (right) Same presentation for the trimer interface in the L171 variant. **C** BabA sequence diversity flanking the 198-199 signature position in selected generalist and specialist strains. **D** Structural features of BabA in generalist and specialist strains. (left) BabA from generalist strain 17875 in complex with blood group B Lewis b heptasaccharide BLe^b^7 (PDB:5F7W). BLe^b^7 is shown as space-filled presentation and BabA as transparent surface. The protein backbone is indicated by a white ribbon with the CL2 and DL1 loops in yellow and orange, respectively. (middle) BabA glycan binding site in the generalist strain 17875 highlighting the position of residues S198 and K199. (right) BabA glycan binding site in the specialist strain S831 (PDB: 5F8R). BLe^b^7 was modelled into the glycan binding pocket to illustrate the steric overlap between the L198 sidechain and BLe^b^7 (magenta arrow) that impedes binding of BLe^b^7

A recent study of over 1,000 *H. pylori* genomes of worldwide origin identified a TCA/TTA SNP in the serine protease gene *htrA* that was significantly associated with the frequency of gastric cancer [17]. This SNP, which codes for a serine/leucine polymorphism at amino acid position 171 (S/L171) in the protease domain of the enzyme (Fig. 1A), was found to affect the proteolytic activity of HtrA [17]. Protein modeling of the HtrA homotrimer, which is the proteolytically active form of the enzyme [19–21], revealed that the amino acid at position 171 is located at the intersection point of all three subunits and interacts with an arginine at position 32 (R32) of the second subunit and an histidine at position 46 (H46) of the third subunit (Fig. 1B) [17, 19]. While S171 from one subunit mainly interacts with R32 from the second subunit, it does not interact extensively with H46 from the third subunit, even though that H46 is also in close spatial proximity (Fig. 1B). In contrast, the bulkier sidechain of L171 not only facilitates strong interaction with R32 from the second subunit, but also closely binds to H46 from the third trimer subunit (Fig. 1B). Thus, the SNP increases the structural stability of HtrA trimers, which directly affects the proteolytic activity of the enzyme and accordingly exhibits important consequences for pathogen-host interactions [17]. In addition to the R32- H46-S/L171 interaction, trimer stability is pH-dependent with a trimer-to-monomer ratio that is increasing with the pH (pH 3 → pH 5 → pH 7) [22]. Enhanced proteolytic activity through increased trimer formation amplified the destabilization of the gastric epithelial layer by cleavage of cell junction proteins, including the tight junction proteins occludin and claudin-8, as well as the major component of the adherens junctions, E-cadherin [17]. Functional studies showed that amplified cleavage of E-cadherin increased the release of β-catenin from the E-cadherin/β-catenin complex. Dissociated β-catenin subsequently relocated to the nucleus, where accumulated β-catenin elicited cell proliferation [17]. In addition to increased damage of the epithelial layer, 171L HtrA-possessing strains induced stronger translocation of *H. pylori* oncoprotein CagA into epithelial cells and triggered more transcription factor NF-κB-mediated and interleukin-8 (IL-8)-mediated inflammation of the gastric epithelium. Moreover, infection with *H. pylori* possessing L171-type HtrA caused a higher rate of DNA double strand breaks in host chromosomes, leading to mutations and genome instability, than infection with strains possessing S171-HtrA [17]. Together, these changes promote the development of severe disease in response to an infection with *H. pylori*, including gastric cancer, and indicate that L171-HtrA is much more destructive to the epithelial cell layer that S171-HtrA.

Which variant is the ancestral HtrA allele in *H. pylori*, the aggressive L171 HtrA or the comparatively weak S171 HtrA allele? Analyses of *htrA* sequences from other *Helicobacter* species revealed that all gastric *Helicobacter* species exclusively possess L171-HtrA. Likewise, *H. cinaedi*, *H. hepaticus*, and 24 different *Campylobacter* species only possess L171, but not S171, whereas other *Campylobacter* and enterohepatic *Helicobacter* species possess other amino acid residues at this position. Given that *H. pylori* is the only species that contains both L171-HtrA and S171-HtrA, the data indicate that the L171 is ancestral [23]. Thus, the milder S171 allele evolved after the ancestral and more aggressive L171 allele, which suggests that the more benign S171 HtrA variant may represent an evolutionary adaptation of *H. pylori* serine protease HtrA that exhibits a reduced impact on the human host during pathogen-host interactions at the epithelial layer. As such, this attenuated HtrA version may promote long-term colonization by the gastric pathogen while inflicting less damage to the health of the infected individual [23]. But when did the less virulent S171 HtrA evolve? Based on whole genome and housekeeping gene sequences *H. pylori* strains are assigned to biogeographic populations that were according to their main geographic source named hpAfrica1, hpAfrica2, hpNEAfrica, hpEurope, hpEastAsia, hpAsia2, hpNorthAsia, and hpSahul [24–26]. The distribution of L171 and S171 HtrA alleles showed that all *H. pylori* biogeographic populations possess both L171 and S171, with the exception of the African populations hpAfrica1, hpAfrica2 or hpNEAfrica that only have L171 HtrA, which indicates that the S171 mutation must have arisen in Asia after the out-of-Africa migration around 60,000 years ago, but before the separation of the hpSahul population from the ancestor of the hpAsia2, hpEastAsia and hpNorthAsia *H. pylori* populations ca. 45,000 years ago [23].

### A similar S/L SNP in the major *H. pylori* blood group antigen adhesin BabA

BabA is another example among *H. pylori* proteins, in which a single nucleotide substitution can significantly affect pathogen-host interactions, particularly binding to the gastric epithelium [18]. Similar to the S/L SNP in HtrA, BabA contains a single C/T nucleotide substitution that results in an S/L change. As pointed out above, BabA binds specific host glycan structures known as ABO/Leb blood group antigens, and this S/L exchange at position 198 in its carbohydrate binding domain (CBD) alters the recognition of blood group antigens [18]. Based on their glycan binding preferences, strains are classified as either specialists or generalists. Specialist strains bind only the shorter blood group type O antigens, whereas generalist strains additionally recognize the longer blood group A and B antigens. Worldwide, more than 95% of the extant *H. pylori* strains are generalists, whereas 60% of strains from South American Amerindian are specialists, which coincides with the unique predominance of blood group O in these Amerindians [9].

Comparison of the CBD sequences revealed that amino acid residue 198 is highly indicative for the glycan binding properties. This is exemplified for a subset of *H. pylori* strains in Fig. 1C. Generalist strains predominantly contain a serine at position 198, whereas in specialist strains, position 198 is occupied by residues that exhibit a bulkier sidechain than serine, e.g. aspartic acid, asparagine, or leucine. With few exceptions, consideration of this protein position allows to classifying *H. pylori* strains into generalists and specialists [18]. In contrast, amino acid position 199 is selected for binding affinity and for acid sensitivity of BabA binding [27].

Crystal structure analysis of representative BabA isoforms in complex with blood group glycans [18] revealed that the BabA glycan binding site is mainly formed by three segments of the peptide chain: the highly conserved cysteine-bridged loop CL2 and the two diversity loops (DL1, DL2). The orientation of residue 198 at the N-terminus of DL1 plays a crucial role for the discrimination between A, B, and O blood group glycans. In the generalist structure, the S198 sidechain is oriented away from the glycan binding pocket, which allows binding of long BLe^b^7 glycan (Fig. 1D). This orientation cannot be adopted by L198 in the specialist strains, which results in a rotation of the bulky L198 into the binding pocket, thereby hampering binding of the blood group A and B glycans and only permitting binding of the smaller blood group O type glycan.

Screening for spontaneous mutants in the *babA* gene resulted in the identification of clones that contained a single base substitution in codon 198 which resulted in a L198S substitution, and thus the switch from a specialist to a generalist phenotype [18]. The ability of *H. pylori* to switch between specialist and generalist phenotypes by a single point mutation (TTG/TCG for L/S or AAC/AGC for N/S) in *babA* may be beneficial for *H. pylori* in human host populations where blood group A and B individuals are scarce, such as in South America before the arrival of Europeans. In populations where blood groups A and B are common, however, generalist strains are at an evolutionary advantage, which selects for generalist strains.

### Other *H. pylori* SNPs with functional characterization

In addition to the above discussed *H. pylori* SNPs, two mutations in the EPIYA-B motif of CagA in Western isolates have been described that result in an A/T amino acid polymorphism with EPIYA and EPIYT variants, respectively [28]. Interestingly, the EPIYT variant is significantly less associated with gastric cancer than the EPIYA variant in motif B. Functional studies have shown that this polymorphism regulated the tyrosine-phosphorylation-dependent interaction of CagA with phosphoinositol-3-kinase (PI3K). The phosphorylated EPIYT variant revealed a higher binding affinity to PI3K, which upregulated AKT kinase activity, and exhibited attenuated production of IL-8 and cell scattering during infection of human epithelial cells, compared to an isogenic EPIYA strain at motif B [28]. These findings suggest that the EPIYA/EPIYT polymorphism controls the functional activity of CagA by de-regulating PI3K and AKT signal transduction associated with gastric cancer development. Another gene polymorphism has been detected in the *H. pylori* iron uptake regulator Fur [29]. A single SNP (R88H) in Fur was found to occur during experimental infection of Mongolian gerbils, and isogenic *fur* variant strains revealed that the R88H mutation increased the resistance of the bacteria against oxidative stress during infection of neutrophils and high salt stress, thus providing an evolutionary advantage [29]. Furthermore, genome sequencing of input and output *H. pylori* strains during infection of humans, rhesus macaques and gerbils identified elevated mutation rates in the bacteria and the occurrence of additional SNPs in *H. pylori* genes, whose functional importance was not yet investigated and awaits further characterization [4, 27, 29]. Likewise, the functional importance of two further SNPs in serine protease HtrA that correlated with gastric cancer, namely an aspartate/glutamate change at amino acid position 39 (allele E39), and a valine/isoleucine substitution at position 439 in the PDZ2 domain (allele I439), remains to be elucidated [17]. Finally, three genome-wide association studies (GWAS) identified *H. pylori* SNPs epidemiologically associated with gastric cancer [14–16]. In some cases, three-dimensional modeling of resulting proteins was performed [15, 16], but functional studies on these SNPs during infection are widely missing. The importance of these and other *H. pylori* SNPs should be investigated in detail in future studies.

## Conclusions

SNPs cause a large part of the genomic variability. Yet, there is a significant lack of understanding how individual microbial SNPs influence the interactions between a pathogen and the cells and tissues of its host. Recent studies started to accumulate data on how single amino acid substitutions alter protein properties in bacterial virulence factors, and how these changes modify the way or intensity of the interaction at the pathogen-host interface. The S/L171 mutation in HtrA drastically changes the efficiency at which this protease destroys structural proteins of the cell-to-cell connections in the host’s gastric epithelium. The S/L198 change in BabA determines whether this adhesin binds all three A, B and O blood group antigens with high efficiency or is limited to binding of the blood group O antigens. These detailed functional studies discovered how a serine/leucine substitution resulted in markedly different protein characteristics and how such a small change had drastic effects on the outcome of the pathogen-host interactions. This is particularly important in regard of the associated disease manifestations, as the L171 allele was found to be strongly associated with the development of gastric cancer. As such, additional cancer-related SNPs were identified in *htrA* and in several other *H. pylori* genes that await detailed functional characterization to unravel the mechanisms of how a single amino acid substitution strongly increases the risk of disease.

## Data Availability

No datasets were generated or analysed during the current study.
